# *In vivo* label-free tissue histology through a microstructured imaging window

**DOI:** 10.1063/5.0165411

**Published:** 2024-01-09

**Authors:** Claudio Conci, Laura Sironi, Emanuela Jacchetti, Davide Panzeri, Donato Inverso, Rebeca Martínez Vázquez, Roberto Osellame, Maddalena Collini, Giulio Cerullo, Giuseppe Chirico, Manuela Teresa Raimondi

**Affiliations:** 1 Department of Chemistry, Materials and Chemical Engineering “Giulio Natta,” Politecnico di Milano, Piazza L. da Vinci 32, 20133 Milan, Italy; 2 Department of Physics, Università di Milano-Bicocca, Piazza della Scienza 3, 20126 Milan, Italy; 3 Division of Immunology, Transplantation and Infectious Diseases IRCCS San Raffaele Scientific Institute, Vita-Salute San Raffaele University, Milan, Italy; 4 Institute for Photonics and Nanotechnologies (IFN), CNR and Department of Physics, Politecnico di Milano, Piazza L. da Vinci 32, 20133 Milan, Italy

## Abstract

Tissue histopathology, based on hematoxylin and eosin (H&E) staining of thin tissue slices, is the gold standard for the evaluation of the immune reaction to the implant of a biomaterial. It is based on lengthy and costly procedures that do not allow longitudinal studies. The use of non-linear excitation microscopy *in vivo*, largely label-free, has the potential to overcome these limitations. With this purpose, we develop and validate an implantable microstructured device for the non-linear excitation microscopy assessment of the immune reaction to an implanted biomaterial label-free. The microstructured device, shaped as a matrix of regular 3D lattices, is obtained by two-photon laser polymerization. It is subsequently implanted in the chorioallantoic membrane (CAM) of embryonated chicken eggs for 7 days to act as an intrinsic 3D reference frame for cell counting and identification. The histological analysis based on H&E images of the tissue sections sampled around the implanted microstructures is compared to non-linear excitation and confocal images to build a cell atlas that correlates the histological observations to the label-free images. In this way, we can quantify the number of cells recruited in the tissue reconstituted in the microstructures and identify granulocytes on label-free images within and outside the microstructures. Collagen and microvessels are also identified by means of second-harmonic generation and autofluorescence imaging. The analysis indicates that the tissue reaction to implanted microstructures is like the one typical of CAM healing after injury, without a massive foreign body reaction. This opens the path to the use of similar microstructures coupled to a biomaterial, to image *in vivo* the regenerating interface between a tissue and a biomaterial with label-free non-linear excitation microscopy. This promises to be a transformative approach, alternative to conventional histopathology, for the bioengineering and the validation of biomaterials in i*n vivo* longitudinal studies.

## I. INTRODUCTION

Biomaterial discovery and engineering are one of the largest and most active topics in biomedical research, with a large and direct impact on the development of implantable prostheses and engineering of artificial tissues.^1,2^ However, clinical and regulatory norms strictly regulate the development and use of implantable biomaterials, whose costs and validation *iter* are one of the major limiting factors for a further progress in the field. As an example, the estimated cost for the validation of new biomaterials can span the range €60 000–€120 000 per product, depending on the final device invasiveness.^1,3^ Therefore, there is currently a large interest in new techniques and methods aimed at the validation of biomaterials in a less expensive way. This goal can be reached by reducing the working time of highly specialized personnel and by reducing the number of employed laboratory animals, which has a large ethical impact.

Procedures for the biomaterial validation are defined by the ISO10993 norm^4^ that relies on the visual inspection of tissue sections to score any evidence of an undergoing foreign body reaction (FBR), like the recruitment of immune cells.^5^ This preclinical research step requires the sacrifice of a huge number of laboratory animals, contradicting the basic 3R's principles^6^ with an unsustainable ethical burden. Other characteristic signs of FBR, such as angiogenesis, collagen I–III deposition, and fat infiltration, are also analyzed on tissue sections from laboratory animals with protocols (primarily immune-histochemistry), which are over 40 years old.

Therefore, it is highly relevant to develop inspection methods that allow for a more complete and real time analysis of the vascular capillaries,^7^ providing invaluable information on the biocompatibility of a medical device, well beyond the current ISO 10993-6 norms.

Recent scientific advances have consistently and effectively demonstrated the possibility to directly visualize in long-lasting observations the recruitment of the immune cells^8^ and the related fibrotic reaction *in vivo* by means of non-linear excitation microscopy.^9–12^ Similar intravital inspections would be extremely valuable to test biomaterials,^9,10^ greatly improving the quality and reliability of the assessment of the reaction to implants.^13^ To achieve this aim, significant advances in deep tissue optical microscopy are necessary. Although invasive skull intravital microscopy techniques have been largely demonstrated,^14,15^ through-skin optical imaging would have high potential to reduce observation invasiveness, but it is still challenging. A part from the possibility of optical clearance of the skin,^16^ one can try to overcome this limitation by implanting an optical window in the animal, also known as window chamber (for example, cranial or dorsal), to avoid passing through the dense skin *stratum corneum*.^14^ Among the variety of existing imaging windows developed so far,^14,17^ one of the most used ones is the dorsal skin-fold chamber.^18,19^ As an example, Dondossola *et al.*^9^ presented a detailed high-resolution visualization of the reaction to samples of electrospun fibers subcutaneously implanted in mice for up to 14 days. More recently, the cytotoxic effect of the topical release of anticancer drugs by tiny micro-dispensers (volume $≅4\times 0.8\times 0.8\;{\mathrm{mm}}^{3}$) implanted in mice was visualized *ex vivo* and *in vivo*.^20^ In this case, Jonas and colleagues used either endoscopic confocal^21^ or two-photon excitation fluorescence (TPEF) microscopy^22^ to image the response of cancer cells to drugs released locally at the tumor site.

The application of the window chamber concept to immune reaction of implants using high resolution optical microscopy would require a thorough analysis of the possibility of identifying immune cells and microvessels at the implant site *in vivo* or on fresh biopsies, to replace the time-consuming procedure of histological analysis on thin tissue sections. The relevant questions are basically two. Is it possible to perform label-free high-resolution microscopy of tissue *in vivo* replacing histology analysis? Is it possible to follow the histological changes in longitudinal studies *in vivo*? To address these issues, we should prove that non-linear excitation microscopy provides high resolution cellular details *in vivo* without the need of tissue staining. We should also be able to prove that highly porous microstructures implanted in lab animals offer a reliable frame of reference for repeated, longitudinal observation of the tissue dynamics.

Here, we performed a direct comparison of H&E histopathology to non-linear optical imaging *in vivo* of a microstructure, called Microatlas,^23^ implanted in the chorioallantoic membrane (CAM) of chicken embryos and observed label-free. The Microatlas is a photopolymerized microstructure composed of a highly regular microscaffold, consisting of a rectangular lattice, with the dimension of $50\times 50\times 20\;\mu m^{3}$, which is infiltrated by CAM cells when implanted. The micro-structure includes several features, which act as beacons for its 3D orientation and for the optical allignment of the device under a microscope for longitudinal studies. It is fabricated in a biocompatible resin, typically used for dental applications,^24^ mildly doped with a photosensitizer. The polymerized resin is fluorescent under non-linear excitation, and it can be, therefore, visualized in microscopy images without hindering the visualization of the growing tissue. As we will show in this contribution, the choice of the geometrical structure allows a good infiltration of tissue and vessels while keeping a good resistance to the bio-mechanical stresses induced by the growing tissue, at least in the chicken embryo animal model employed here.

We further show that cell type recognition and quantification of their density and shape can be obtained by non-linear excitation optical imaging *in vivo*, perfectly matching the standard H&E histopathology analysis performed on tissue fixed sections *ex vivo*. Second harmonic generation (SHG) microscopy on fresh tissue CAMs also allows us to ascertain the presence and geometrical features of the micro-vessels in the Microatlas.

## II. RESULTS

### A. Geometry of the Microatlas device

**FIG. 1.**
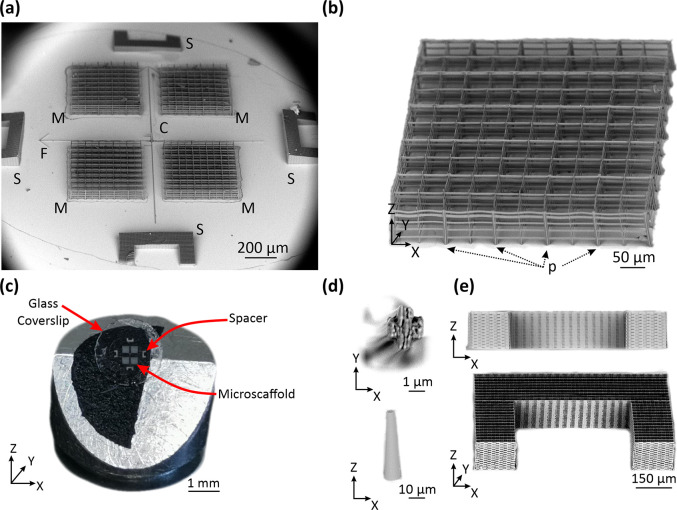
Description of the implantable device constituted by four Microatlas structures and spacers. Panel (a) reports a low magnification scanning electron microscopy (SEM) micrograph of the whole device with indications of the Microatlas (M), the spacers (S), the frame of reference (F), and the reference cone (C). Panel (b) reports a SEM micrograph of one Microatlas lattice, with the details of the thicker pillars (P) and the microgrid. Panel (c) is a photograph of a whole device composed of four Microatlas (M), four spacers (S) on a 5 mm diameter glass slide. Panel (d) reports details of the pillar's cross section and the reference cone. Panel (e) reports a cross section (x–z) view and a perspective view (xyz) of one C-shaped spacer.

The whole micro-structured device carries four identical Microatlas grids [M in Fig. 1(a)] each being realized as a rectangular lattice [Fig. 1(b)] fabricated by two-photon polymerization (2PP) in the SZ2080 biocompatible resin, on the surface of an optically transparent substrate, a borosilicate circular glass [Fig. 1(c)]. The porous microstructure, obtained by means of tightly focused ( ≃ 1 μm) beams, can be chosen arbitrarily in the fabrication process: the overall regular structure of the Microatlas can extend for hundreds of micrometers in the fabrication plane and for about 100 *μ*m along the optical axis [perpendicular to the plane of the substrate, Fig. 1(b)]. Here, we adopted a pore size of about 50 μm for the lattice unit of the Microatlas to allow angiogenesis: each pore of the Microatlas has an inner rectangular size of 49.6 × 49.6 × 20 *μ*m^3^ (SI1. “Microatlas design and parallel two-photon polymerization protocols”). At the same time, the structure rigidity should not be decreased by the enhanced porosity (larger pore size) in order to stand the stresses induced by the tissue growing inside. This can be achieved by inserting sparse thicker pillars in the structure that increases its structural stability. With the aim of future massive exploitation in *in vivo* implants, we first focused our efforts on keeping the fabrication time of this highly porous implantable structure low, and on testing what was the reaction of the CAM to its implant.

### B. Fast prototyping of rigid microstructures with high porosity

Our strategy was to insert square-shaped pillars [P in Fig. 1(b)] 3–4 times thicker than the rest of the Microatlas grid elements interleaved every 100 *μ*m (i.e., every two pores) and with an X-shape cross section [Fig. 1(d)]. In this way, we were able to obtain stable scaffolds for up to 4 days after the implantation and to mitigate the increase in the fabrication time due to the larger volume to be polymerized. Moreover, the X-shaped cross section of the pillars helps in minimizing their fluorescence signal contribution to the images. The fluorescence of (the photosensitizer within) the polymerized resist can be induced by two-photon excitation at 800 nm (Ref. 23) with a two-photon action ( ≃1.1 GM)^25^ that is ten time larger than that of redox plasmatic enzymes like NAD ( ≃0.15 GM). However, the typical concentration of the enzymes is about ten times larger ( ≃500 μM) than that of the photosensitizer in the resist ( ≃33 μM), resulting in a very similar level of fluorescence emission. The X-shaped cross section of the pillars helps also in further reducing the fabrication time, while, at the same time, providing high rigidity to the pillars. The pillars X-shape cross section had a 5 μm side and a 1.5 *μ*m arms' thickness. These structures [Fig. 1(d)] were fabricated with laser writing speed of 2.5 mm/s, and voxel size R×R×Z=0.35×0.35×1 μm^3^. The resulting 36 pillars, interleaved in the Microatlas grid, were then realized four at a time thanks to a four “square” mask entailing a parallel fabrication with overall process time of 1.50 min (see Table SI1.1).

In order to minimize the fabrication time per device, the laser 2PP writing of the device was implemented in parallel mode, by coupling the optical scanning setup with a spatial light modulator (SLM) to generate multiple laser foci.^26^ This process (see SI2, “Two-photon laser polymerization setup and parallel two-photon polymerization protocol”), assisted by a smart choice of the beam spots array for the fabrication of the various features of the device, considerably increased the process throughput,^26^ with a total fabrication time per Microatlas of 2.5 min (see SI2, “Microatlas design” and in particular, Table SI1.1). This was achieved with a X–Y inter-voxel spacing R = 0.35 *μ*m as the optimal choice leading to stable and perfectly reproducible lattices, with X–Y writing speed of 3 mm/s. The writing speed along the Z direction was lowered to 1 mm/s to reduce vibrations during the polymerization process. The final device for implant is constituted by four porous Microatlas grids (M), four dense spacers (S), and several reference objects [F, C indicated in Figs. 1(a) and 1(d)]. Since the fabrication time scales with the third power of the object size,^27^ also the geometrical parameters of these supporting additional elements were carefully chosen. The most demanding elements in terms of fabrication time were the dense spacers [Fig. 1(e) and S in Fig. 1(a)], with a total side of 500 *μ*m and horn length of 150 *μ*m. These dense microstructures (tight woodpile polymerization pattern with lattice unit = $10\times 10\times 2\;\mu m^{3}$) needed a fabrication time of about 30 mins with a speed of 3 mm/s (see Table SI1.1). Finally, the entire device fabrication was based on a total of six phase masks with a total fabrication time of about 47 min and a time saving of 70% with respect to the single-focus fabrication with the same laser spot geometry. Notably, the fabrication time increases only by 6% per each additional scaffold (Table SI1.1).

### C. Visualization of Microatlas implanted in chicken embryos allows cell density evaluation

**FIG. 2.**
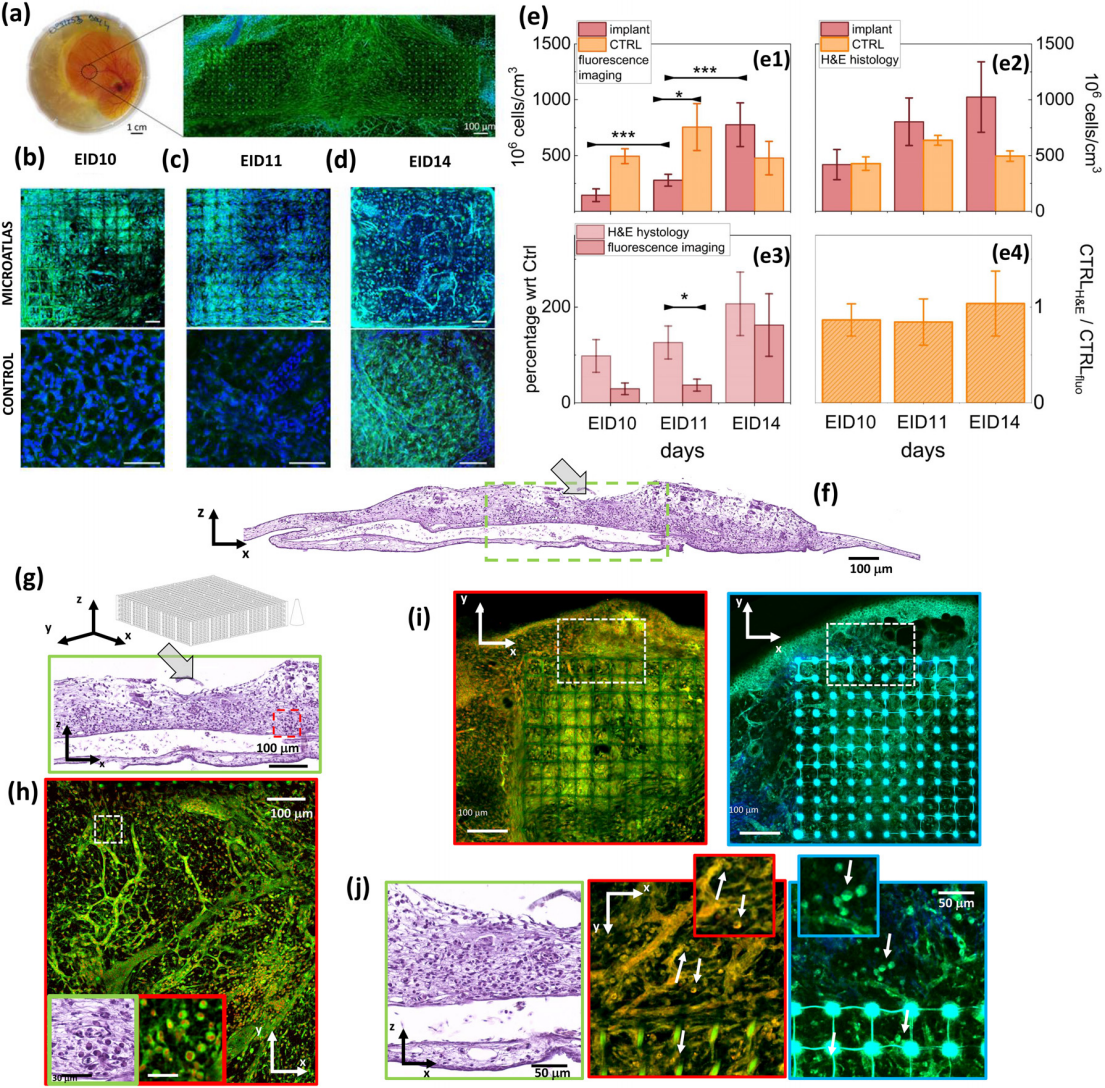
Evaluation of the cellular density. Microatlas implanted *ex ovo* in CAM and comparison of H&E histology and of fluorescence microscopy analysis. In panel (a), we show the implants in the embryo and a wide field of view of the implant taken under two-photon excitation ( $\lambda_{\text{exc}}$ = 800 nm). Panels (b)–(d) present details of a Microatlas grid, imaged by two-photon excitation, at EID10, EID11, and EID14 (top row, bar size = 50 μm) and of control tissue (bottom row, bar size = 50 μm). Signals are fluorescence from cytoplasm and vessels (green, Exc: 800 nm, Em: 535/50 nm) and second harmonic generation from collagen I (blue, Exc: 800 nm; Em: 400/40 nm). Panels (e): cell counting in the Microatlas and control tissue: (e1) cellular volume density measured on 3D fluorescence images in the Microatlas (dark wine) and in the control samples (light orange); (e2) cellular volume density measured on H&E stained tissue sections measured around the Microatlas (dark wine) and in the control samples (light orange); (e3) percentage increase in cellular density in the Microatlas as measured on fluorescence images (dark wine) and as measured on the H&E sections around the Microatlas (pink); and (e4) ratio of the cell counting on the control samples as measured on the histological sections over the cell counting as measured on the fluorescence images. Panel (f) H&E stained cross section (x–z plane) of a CAM. The pocket in which the Microatlas was implanted is indicated by a arrow. Tissue fixed at EID14. Panels (g) and (h) compare standard H&E staining to confocal images. Panel (g) reports the area framed in panel (f), below the Microatlas implantation site (x–z section), in H&E staining. Panel (h) shows the *confocal image* of the tissue area near [approximately the red boxed area in panel (g)] the Microatlas (the pillars of two microgrids are visible in the upper part of the image). The green and red framed boxes show a zoomed area of granulocytes visualized by means of standard H&E and of confocal microscopy, respectively. Color codes are vessels, cells cytoplasm and Microatlas autofluorescence (Exc: 488 nm, Em: 500–550 nm, green) and nuclear fluorescence due to DRAQ5 (Exc: 633 nm; Em: 645–720 nm, red). Panel (i) illustrates the power of *label-free histology*. Confocal and TPEF images of the tissue surrounding a Microatlas are reported in the left (red framed) panel and in the right (light blue framed) panel. Panel (j) direct comparison of confocal and TPEF high resolution images of the field of view boxed in white dashed frames in panel (i) (x–y plane). Examples of granulocytes visualized by confocal (red framed) and TPEF (light blue framed) images are indicated by white arrows and in the corresponding blow-ups. The green framed box reports the detail of the x–z section corresponding to the confocal and TPEF images. For confocal images, color code is as in panel (h). For TPEF images, blue: second harmonic signal generated from collagen and Microatlas autofluorescence (Exc: 800 nm; Em: 400/40 nm); green: vessels, cells cytoplasm and Microatlas autofluorescence (Exc: 800 nm, Em: 535/50 nm).

**SCHEME 1.**
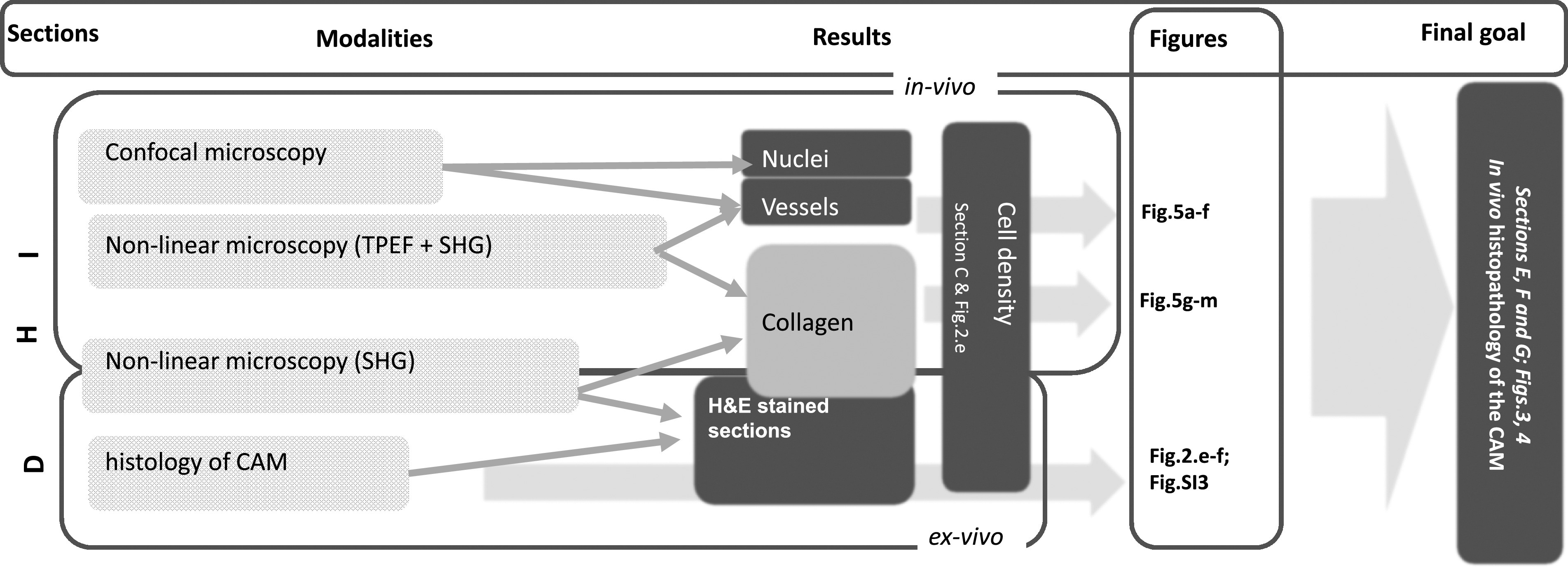
General scheme of the multimodal approach aimed to assess the possibility to perform histological analysis in chicken embryos through *in vivo* non-linear excitation microscopy. CAM = “Chorioallantoic membrane;” TPEF = “two-photon excitation fluorescence;” SHG = “Second Harmonic Generation.” The sections of this paper, the modalities (TPEF, SHG, etc.), the outputs (Results) and the corresponding figures are listed on the top bar of the scheme.

The quantification of the number density of cells is essential for any assessment of the immune reaction to an implant. The Microatlas implanted in living chicken embryos can provide this information. To prove this, our main aim is to show that optical microscopy imaging of the CAM provides enough information also to assess the immune reaction to the implant in terms of recruitment of cells. This is achieved by a thorough comparison of the histological analysis (*ex vivo* imaging on H&E-stained sections) to non-linear excitation microscopy, a very well-established technique for *in vivo* imaging. Confocal fluorescence microscopy on *ex vivo* samples (in which the cell nuclei were stained with the DRAQ5 dye) was also used as a cross-check of the shape and internal structure of the nuclei. *Ex ovo* implantations of the devices [Fig. 2(a)] were performed at Embryonic Incubation day 7 (EID7) following a modified protocol of the well-established CAM assay.^23,28^ We pursued a multimodal study of different aspects of the immune reaction according to the general Scheme 1.

In our *in vivo* experiments, non-linear excitation microscopy was applied without the need of a specific labeling. The SHG signal was used to monitor the formation of collagen type I [Figs. 2(b)–2(d), blue channel] and to quantify the fibrotic reaction. TPEF of endogenous metabolic cofactors was used to single out cells, in particular granulocytes [Figs. 2(h) and 2(j), green channel and white arrows]. In all experiments, we acquired and analyzed images taken on at least three implanted chicken embryos, at 3, 4, and 7 days after the implantation (corresponding to EID10, EID11, and EID14, respectively) and compared these results to those obtained from untreated embryos. The cell density was evaluated *in vivo* on the implanted Microatlas and in control CAMs by TPEF label-free imaging and cross-checked against the analysis of images taken on H&E stained tissue sections and against confocal fluorescence microscopy images taken on *ex vivo* samples stained on the nuclei with the DRAQ5 dye. At first, the cells' nuclei were counted on randomly selected 1 μm thick non-linear microscopy optical sections, finding a substantial stability of the tissue density in the control regions in the time range EID10–EID14 [Fig. 2(e1)]. In contrast, the cell infiltration in the Microatlas implanted in embryos showed a significant increase with the implant duration. The cell density in the Microatlas increases steadily from EID11 with a doubling interval of 1.6 ± 0.4 days in the first 7 days of implant, which can be taken as a clear sign of cell infiltration in the Microatlas. The increase in cell infiltration at EID14 could suggest a more pronounced cell infiltration by B-/T-cells because of the physiological evolution of the reaction to the implant.

### D. *Ex vivo* histopathology of the implanted CAM

Direct histopathological analysis with H&E staining is the gold standard of tissue analysis^29^ and provides detailed information on the tissue state and possible inflammatory reaction to the Microatlas implant. Therefore, to validate the results of the fluorescence microscopy analysis of the cell density in the implants, we performed H&E analysis on 4 μm sections of the CAM membrane [Fig. 2(f) and Figs. SI3.1 and SI3.2], both for controls and implanted embryos in the time windows EID10–EID14. On the H&E-stained cross sections of the control CAM samples, we can clearly identify the main tissue components:^29^ the fibrous chorionic ectoderm, the central mesenchymal layer rich in vessels, and the allantoic endoderm (Figs. SI3.1 and SI3.2). The mesodermal layer of the allantois becomes fused with the adjacent mesodermal layer of the chorion after a few days from fertilization. The vascular network that develops in the mesenchymal double layer and that is connected with the embryonic circulation has a respiratory function and allows the uptake of calcium from the egg shell.^30–32^ Upon implant, the vessels are growing in size [Fig. 2(f) at EID14 and Figs. SI3.1 and SI3.2, at EID11–EID15], indicating the recruitment of cells in response to the implanted Microatlas, initially recognized as a foreign body. Cells were counted on the H&E-stained tissue sections and the cellular volumetric density was estimated close to implant site and in control samples [Fig. 2(e2)] by considering the average nuclear size and the section thickness. The general trend observed on the fluorescence microscopy images taken *in vivo* is replicated here, with a steady increase in the cellular density found close to the implant site corresponding to a doubling interval of 4 ± 2 days in the first 7 days of the implant. In the control samples, as observed on the fluorescence images, the cellular density reaches a plateau level at least from EID10. To estimate the agreement between the H&E and the fluorescence imaging analysis, we have computed the relative increase in the cellular density in implants, with respect to the control tissues, as a function of the incubation time. This relative cellular density increases with time [Fig. 2(e3)], with a doubling interval of 3.5 ± 0.7 days and 2.0 ± 0.8 days for the H&E and the fluorescence imaging analysis, respectively. It is relevant for our purpose to notice that the trend is similar in the two cases and the only significant difference (p ≃0.05) is found for EID11 [Fig. 2(e3)]. On the contrary, the cell density in the control tissues is found to agree between the two methods within 10% [Fig. 2(e4)]. These observations support our hypothesis that the cellular density can be evaluated on the fluorescence images as well as on the histological sections.

**FIG. 3.**
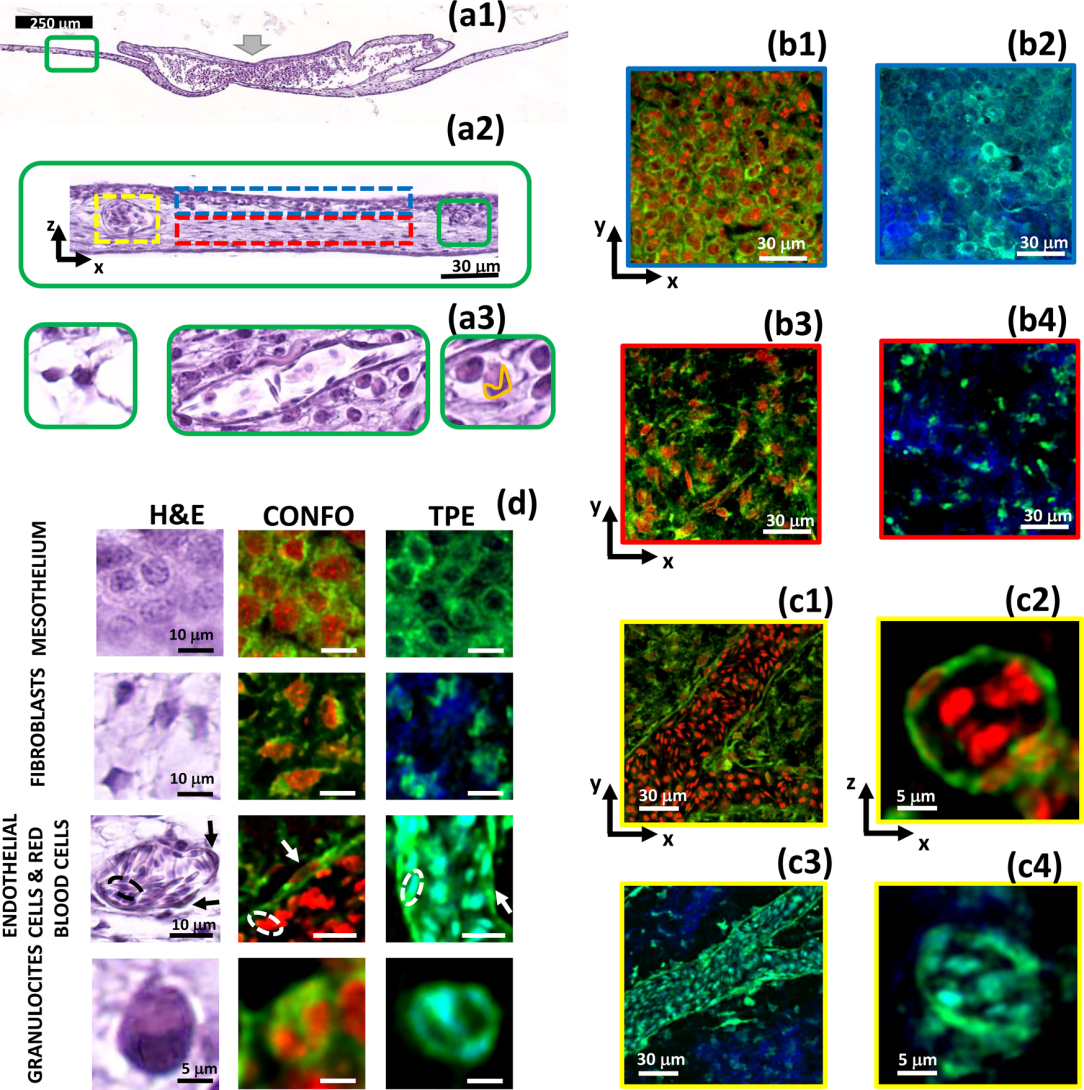
Visualization of the chicken embryo foreign body reaction to the Microatlas implant by H&E histology and fluorescence imaging. Panels (a) reports, at various levels of magnification (coded in colors), the H&E images of a CAM cross section at EID14 of an embryo in which the Microatlas was implanted [the implant site is indicated by the fat gray arrow, panel (a1)]. Examples of mesothelium, chorionic ectoderm, and the vessels are identified by blue, red, and yellow dashed boxes [panel (a2)]. The increasing levels of magnification are identified by green boxes and provide examples of granulocytes (right, whose nuclei are segmented by an orange line), fibroblasts (left), and vessels (center) with nucleated red blood cells [panel (a3)]. Panels (b) and (c): Confocal fluorescence and non-linear excitation images are compared to H&E one [panel (d), first column]. The mesothelium layer, the chorionic ectoderm and the vessels are identified in the H&E image [panel (a2)] by a red, a blue, and a yellow dashed box, respectively. Confocal images [panels (b1), (b3), (c1), and (c2)] are collected on *ex vivo* CAM samples stained with DRAQ5 dye (nuclei, red channel) and exploiting the cytoplasm auto-fluorescence (green). *In vivo* (*ex ovo*) non-linear excitation images were collected upon excitation at λ=800 nm [panels (b2), (b4), (c3), and (c4)]. Green and blue channels are the cell auto-fluorescence and the second harmonic signal coming from the collagen fibers. The color (red, blue, and yellow) of each image edge corresponds to the color of the boxes in panel (a2). Panels (c2) and (c4) report details of the red blood cells within capillaries in confocal fluorescence images (c2) and under non-linear excitation (c4). Panel (d): atlas of single cells as identified on the H&E, the confocal and the non-linear excitation images (TPEF column).

**FIG. 4.**
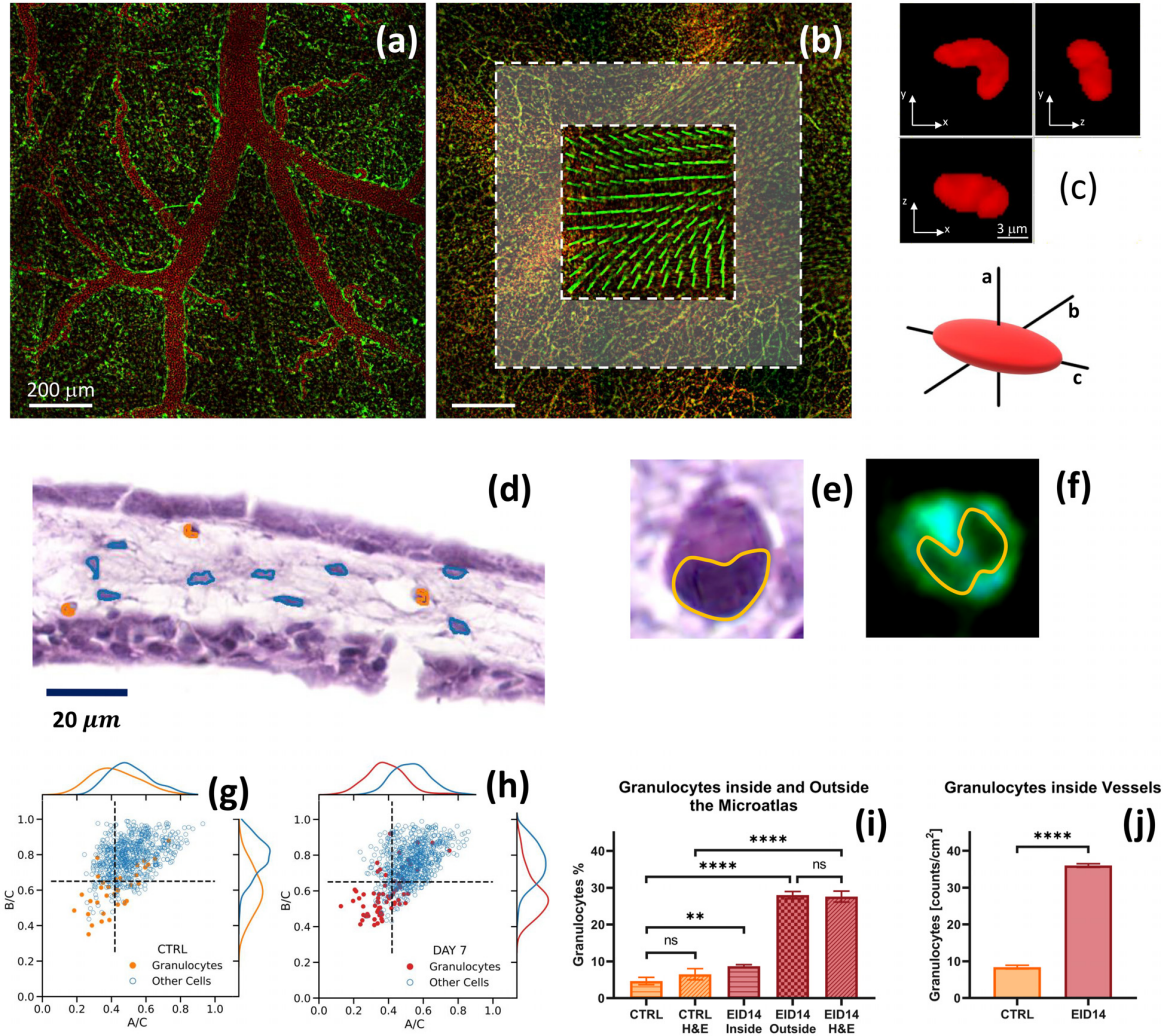
Characterization of the nuclear shape anisotropy. Panel (a): confocal images of control CAM (no implant) taken on a highly vascularized region. Panel (b): confocal image of a Microatlas implanted in a CAM at EID14. Pillars of the Microatlas are clearly visible together with vessels inside (inner dashed framed box) and outside (outer gray box) the Microatlas. Granulocytes were counted in 50 × 50 μ m^2^ ROIs and averaged. Panel (c): three orthogonal cross sections of a granulocyte segmented on a fluorescence microscopy image, together with the definition of the ellipsoid axes. Panel (d): H&E histological image of the x–z section of a CAM (mesothelium) with granulocytes and fibroblasts segmented around the Microatlas, marked in orange and cyan, respectively. Panels (e) and (f) report the detail of a granulocyte whose bilobate nucleus is segmented on the H&E and the TPEF images, respectively. In the TPEF image the nucleus appears dark on the background of the cytoplasmic autofluorescence. Panels (g) and (h): distribution of the axial ratio B/C as a function of A/C for granulocytes compared to the other cells present in the tissue for a control sample [panel (g)] and samples in which the Microatlas was implanted and observed at EID14 in and around the Microatlas [panel (h)]. The dashed lines identify four quadrants in the correlation plot. Panel (i): percentage of the granulocytes counted on the optical microscopy images inside [inner box, panel (b)] and outside [outer gray region, panel (b)] the Microatlas and on the H&E histological sections (outside the Microatlas). Panel (j): density of granulocytes inside and outside the vessels as measured on the optical microscopy images.

### E. *In vivo* histopathology of the CAM

We can push the equivalence of H&E *ex vivo* histology and *in vivo* fluorescence imaging even further, by proving that we can also identify and recognize the cells from their morphology on fluorescence images. To this purpose, we applied both confocal (on stained samples) and non-linear excitation imaging, label-free. On the confocal images, we visualized the cytoplasm through its auto-fluorescence [green channel, Fig. 2(h)] and the nuclei through the DRAQ5 staining [red channel, Fig. 2(h)]. Confocal images display very similar details to those found on the H&E images of the tissue sections [Figs. 2(g) and 2(h)] both in terms of the vessel bed structure [Fig. 2(h)] and of the cells [Fig. 2(h) insets], identified from the autofluorescence of metabolic enzymes.^33^ By means of non-linear excitation fluorescence microscopy, we can pursue a completely label-free approach *in vivo*, exploiting the cell cytoplasm auto-fluorescence and the SHG signal arising from collagen. The major difference with confocal fluorescence imaging is that in non-linear excitation cell nuclei appear dark, having very low autofluorescence, while the autofluorescence of the Microatlas is stronger [Fig. 2(i)]. This signal, however, is not hindering the observation of the cells within the Microatlas [Fig. 2(j)]. Therefore, both in confocal and non-linear fluorescence imaging, we can easily identify the granulocytes from their cell nuclei in the Microatlas pores and outside it [see arrows in Fig. 2(j), zoom inserts]. In the case of non-linear excitation microscopy, we can also easily segment vessels (from autofluorescence) and collagen (from the SHG signal).

Identification of the different types of cells can be done on H&E images [Fig. 3(a)] at high resolution [Fig. 3(a3)], based on the details of the cell and nuclei morphology. Chicken embryo has a number of histological peculiarities with respect to rodents, one of the most widely used animal model for immunological tests. Avian erythrocytes have small nuclei. Among the macrophages polymorphonucleated system (MPS) cells, granulocytes comprise heterophils more than neutrophils. Heterophil granulocytes typically possess nuclei with three lobes, while most eosinophil and basophil granulocytes possess nuclei with two lobes. All these features can be taken into account for immune cell segmentation on fluorescence images. Moreover, fibroblasts, endothelial and red blood cells [see, for example, Fig. 3(a3)] could be also identified from their shape. However, no information on the tissue reconstituted within the Microatlas can be retrieved on the histological images since the Microatlas is ex-planted to cut the tissue sections. Moreover, in histology we are limited to a 2D reconstruction of the cell morphology and longitudinal studies are lengthy. Instead, fluorescence images allow us to distinguish cells equally well [Figs. 3(b1) and 3(b2)]. Cells in the collagenous mesenchymal layer could be identified as fibroblasts due to their characteristic elongated nuclei [Figs. 3(b3) and 3(b4)]. On the vessel endothelium [Figs. 3(c1) and 3(c3)], we could identify endothelial cells from their shallow cytoplasm [Figs. 3(c2) and 3(c4)]. The nucleated red blood cells with their characteristic small nucleus can also be clearly discerned inside the vessels both in cross sections [Figs. 3(c2) and 3(c4)] and in longitudinal sections [Figs. 3(c1) and 3(c3)]. Notably, this analysis can be pursued both on confocal images [on *ex vivo* samples, see Figs. 3(b1), 3(b3), 3(c1), and 3(c2)] and on non-linear excitation images [on *in vivo* samples, Figs. 3(b3), 3(b4), 3(c3), and 3(c4)], both in and around the Microatlas.

Certain types of immune cells can also be identified label-free on linear and non-linear excitation images. As an example, polymorphonucleated cells (heterophil, eosinophil and basophil granulocytes) can be recognized among the other cells from their characteristic shape and their multi-lobed nucleus [Fig. 3(d), bottom row, and Fig. 4]. Nuclear lobes are not present in lymphocytes (T/B cells), nor in monocytes and macrophages. Lymphocytes have highly regular round-to-oval nuclei. Monocytes have kidney-shaped nuclei, without lobes, and macrophages have irregular oval nuclei. Mast cells have round-to-oval nuclei, without lobes. Since polymorphonucleated cells (hereafter identified simply as granulocytes) are more easily discernable and they are the first line of reaction to an implant, we have focus on them in this work. The capability to identify this type of cells, with such a high degree of accuracy, is particularly relevant to ascertain the degree of the immune response. It is particularly noteworthy that similar results can be obtained with confocal fluorescence (nuclei stained with DRAQ5 in *ex vivo* samples) and with two-photon excitation microscopy images *in vivo*, in which case no staining was necessary at all. It is also important to notice that the presence of the Microatlas, with its dim fluorescence signal, does not prevent our morphological analysis but offers a frame of reference for repeated longitudinal studies. These findings open the way to the use of the Microatlas as an implantable platform for the quantification of the immune reaction to a biomaterial, once tight coupling between the two is ensured.

Based on our results, we were able to build a morphological atlas of cells exploiting the direct comparison of H&E, confocal fluorescence and non-linear excitation images, as reported in Fig. 3(d) for fibroblasts, endothelial and red blood cells, and for granulocytes.

### F. Indications of a mild immune reaction to the implant

We can now pass to a more detailed analysis of the FBR as a function of the implant duration. Starting at EID11 we observe hyperemia [Figs. 3(a1) and SI3.2], concomitant with growth of the thickness of the CAM, due to an augmented blood flow carrying granulocytes for a prompt immune reaction to the implant. At EID14, we can recognize fibroblasts and cells from the mononuclear phagocyte system (MPS, comprising granulocytes, and likely also monocytes and macrophages) [Fig. 3(a3)]. We can distinguish them both in confocal and non-linear excitation fluorescence images [Fig. 3(b)] and in the H&E stained sections [Fig. 3(d)]. In fact, fibroblasts can be singled out in the mesoderm [Figs. 3(a3) and 3(d), second row from top], with their characteristic elongated shape of the nuclei. Among the MPS cells, the granulocytes (comprising in chicken embryo, heterophils, eosinophils and basophils) can be highlighted by their smaller and bilobed (heterophils) and trilobed (eosinophils and basophils) nuclei [Figs. 3(a3) and 3(d), second row from bottom]. The marked presence of this kind of cells is indeed an indication that an inflammatory reaction is ongoing at the implant site. It is possible that concomitant to the recruitment of MPS cells at EID14, T- and B-cells are also converging to the implant site, complementing the reaction due to MPS cells. However, the limited deposition of collagen I [Figs. 3(b2), 3(b4), 3(c3), and 3(c4)] that we observe through the SHG signal (blue channel) in correspondence of the implant region is a clear indication that the reaction is not massive. Moreover, no indication of necrosis and calcifications is visible at all.

### G. Shape and size of cells' nuclei in the Microatlas

High resolution fluorescence microscopy allows us also to fully characterize the geometry of cell nuclei in the capillaries [Fig. 4(a)], in the tissue around [Fig. 4(b), gray box] and inside the Microatlas [Fig. 4(b), inner dashed box]. In this way, we can gain additional information for the identification of the type of cells directly on the fluorescence images. To this purpose, infiltrated cells, and more specifically cell nuclei, can be randomly isolated and segmented and the morphological features of their nuclei can be characterized by force fitting their surface to a 3D ellipsoid with axes,^34^ A≤B≤C [Fig. 4(c)]. This simple description can capture the major differences in the nuclear shape, like the one of the granulocytes for which the nuclei appear bilobate [Fig. 4(c)] and can be approximated by a prolate ellipsoid. The recognition of the nuclear shape anisotropy can be performed on images of histological sections stained with hematoxylin an eosin [Figs. 4(d) and 4(e)] and on non-linear excitation fluorescence microscopy images, in a label-free approach [Fig. 4(f)]. The distributions of the three axes (Fig. SI4) of the nuclei segmented on the control tissue can be described by a single Gaussian function with average values: ${\left\langle A\right\rangle }_{\mathrm{CTLR}}$ = 1.4 ± 0.4 $\mu m,\;{\left\langle B\right\rangle }_{\mathrm{CTLR}}$ = 2.2 ± 0.4 $\mu m,\;{\left\langle C\right\rangle }_{\mathrm{CTLR}}$ = 2.9 ± 0.6 μm for the control samples, and ${\left\langle A\right\rangle }_{\mathrm{EID}14}$ = 2.3 ± 0.4 $\mu m,\;{\left\langle B\right\rangle }_{\mathrm{EID}14}$ = 3.12 ± 0.5 $\mu m,{\left\langle C\right\rangle }_{\mathrm{EID}14}$ = 4.26 ± 0.6 μm for the Microatlas implanted at EID14. Systematically larger values are found for the embryos in which the microstructures were implanted (see SI4, “Analysis of the shape anisotropy of cell nuclei in CAM”).

Interestingly, we find a correlation between the different axes (see Fig. SI4, off diagonal plots) that suggests the use of the axial ratios A/C and B/C as good parameters for identifying the cells in terms of their nuclear shape anisotropy. Large anisotropies fall in the third quadrant of the plot of B/C as a function of A/C. Indeed, granulocytes show a markedly higher anisotropy compared to all the other cells in control samples [Fig. 4(g)], with low values of the B/C as and A/C ratios. This is even more evident in implanted embryos as shown for EID14 in Fig. 4(h). Most of the granulocytes identified in implant regions are confined in the third quadrant of the B/C vs A/C correlation plot [Fig. 4(h), dashed lines]. This and similar findings, that can be derived from high-resolution label-free optical images, can be the basis for an automated algorithm for the segmentation of granulocytes, in longitudinal studies of living avian embryos.

The granulocytes are not uniformly distributed in the tissue. They occur in larger density in a region close [within 200 μm from the microstructure, outer dashed box in Fig. 4(b)] to the Microatlas. The fraction of granulocytes inside the Microatlas is almost $\frac{1}{4}$ of the total granulocytes number measured outside it, a result that is confirmed by the analysis of the images of the H&E stained samples [Fig. 4(i)]. Also in blood vessels, the concentration of granulocytes is found to increase substantially, 4.5 ± 0.1 times [Fig. 4(j)], for implanted samples at EID14 as compared to the control samples. These phenomena can be considered a normal reaction to the injury on the CAM upon implant and do not indicate a massive reaction to the Microatlas itself.

### H. Vascularization in the Microatlas

**FIG. 5.**
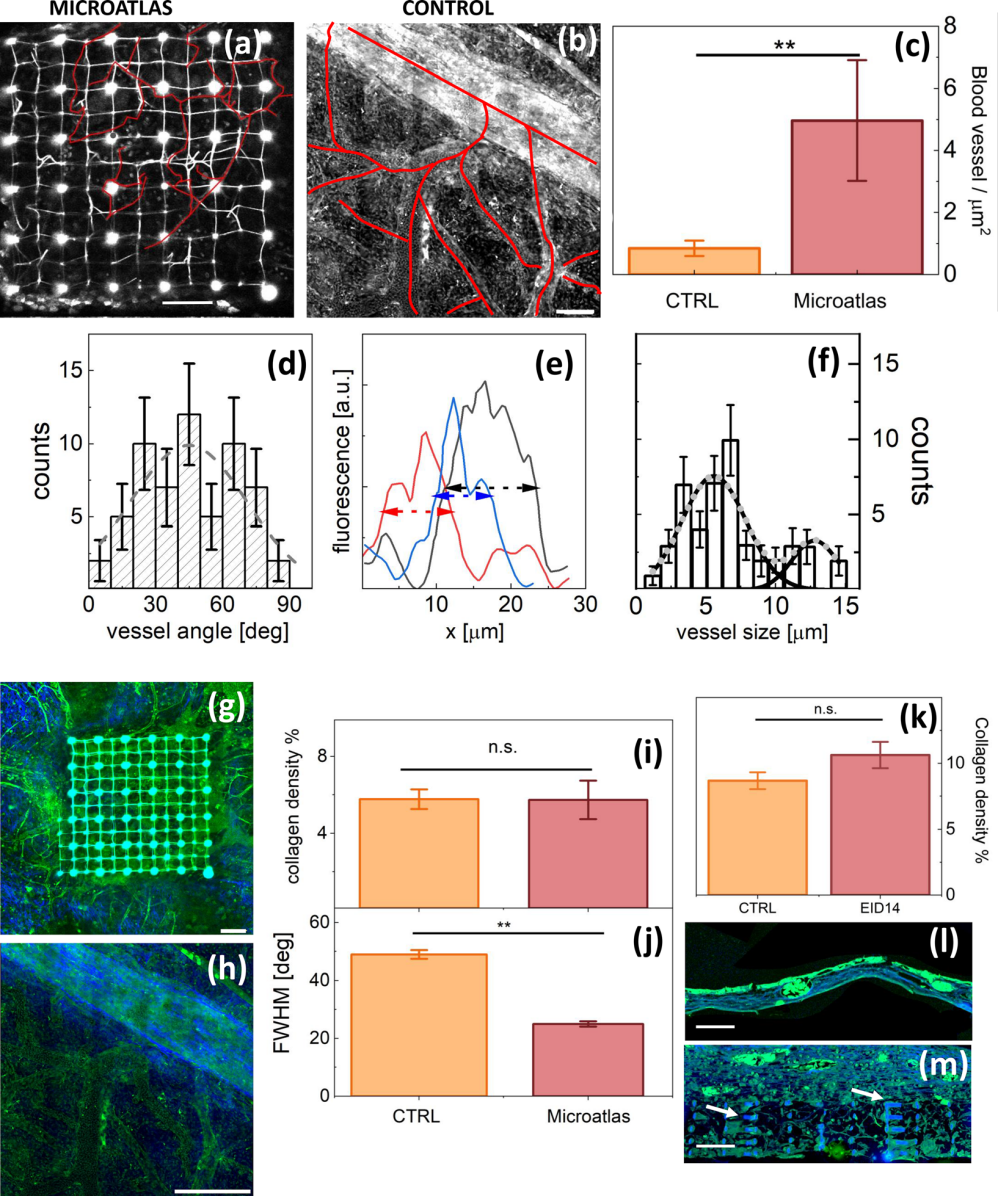
Quantification of the angiogenesis and fibrotic reaction within and around the Microatlas at EID14 on non-linear excitation images. Panels (a) and (b): autofluorescence images of a Microatlas implanted in an embryo [panel (a)] and of a control embryo [panel (b)]. Segmented vessels are highlighted with red continuous lines. Bars are 100 μm[panel (a)] and 50 μm [panel (b)]. Panel (c): vessel density in Microatlas and control tissues. Panel (d): analysis of the orientation of the vessels within the Microatlas. The orientation angle is measured with respect to the side of the Microatlas lattice. The dashed line is a Gaussian fit to the data with a best fit Gaussian FWHM = 60° ± 10°. Panel (e): examples of cross section profile of microvessels in the Microatlas drawn on 2D autofluorescence images [panel (b)]. Dashed lines are full width at half maximum (FWHM). Panel (f): distribution of the vessels' cross section FWHM. The lines are the best fit of the data to a two components (solid black lines, single components, dotted gray line, full fit) Gaussian function with best fit vessel size of 5.6±1 and 12.8±3.5 μm with FWHM = 5.4±1.4 and 3.8±2 μm, respectively. Panels (g) and (h): non-linear excitation images acquired at EID14 (blue: SHG; green: TPEF autofluorescence) of a Microatlas scaffold and a control tissue, respectively. A large ( ≅75 μm cross section) vessel is clearly visible, with further ramifications in the second case. Bars are 100 μm in both panels. Panel (i) reports the density of the collagen fibers as from SHG imaging *in vivo*. Panel (j) reports the values of the FWHM of the angular distribution of fiber orientation angle as derived from Fourier analysis of the images. Panel (k) reports the evaluation of the collagen density on the H&E stained tissue sections as done by counting the content of the pixels in the blue channel (SHG). Panels (l) and (m): non-linear excitation microscopy images where the green and the blue channels correspond to the emission of the Eosin-stained cells and the collagen I fibers, respectively. Bars indicate 100 μm size on both panels. Arrows in panel (m) indicate the presence of residuals of the explanted Microatlas.

Neo-vascularization inside the Microatlas scaffolds [Fig. 5(a)] and in control samples [Fig. 5(b)] can be effectively followed by means of non-linear excitation microscopy (autofluorescence from the metabolic enzymes in the cytoplasm) and characterized in terms of cross section, length and branching ratios. Since the CAM has a very dense capillary network, it is commonly used to study *in vivo* angiogenesis as a response to a foreign body.^35,36^ Vessels grown in implanted embryos, around and within the scaffolds, can be mainly categorized as microcapillaries [compare for example Fig. 5(a)–5(b)]. Their surface density in the Microatlas scaffolds reaches about six times the value measured in the control samples [Fig. 5(c)]. The distribution of the microvessels orientation angles is very wide (best fit Gaussian FWHM = 60° ± 10° ), indicating that there is not a marked preferential orientation with respect to the axes of the Microatlas [Fig. 5(d)]. The microcapillaries average size within the scaffold, estimated from the analysis of their 2D cross sections [Fig. 5(e)], is 7.2±1.5 μm[Fig. 5(f)], less than one half the one measured in control samples that is 18 ± 6 μm. Notably, the distribution of the microvessels within the Microatlas does also show the presence of a second, minor, component with larger vessels FWHM, ≃12.5±2 μm[Fig. 5(f)], that is 70±30% the value of newly formed vessels in the control tissue. The average value of number of branches of microvessels within the Microatlas is 9±2.4, with an average length per branch of 60±11 μm.

### I. Collagen distribution in implants

SHG is a second effective contrast parameter specific for the collagen I in tissue that we can exploit on non-linear excitation images. This parameter allows us also to directly visualize the collagen deposition within the Microtlas scaffold [Fig. 5(g)] and in control samples [Fig. 5(h)] *in vivo*. The formation of collagen I observed in implant regions *in vivo* is not more massive than the one quantified in control regions [Fig. 5(i)], a result confirmed on the TPEF and SHG images of the H&E-stained tissue sections [Figs. 5(k)–5(m)]. Therefore, we have no evidence of the formation of a fibrotic capsule around the Microatlas implant. The orientation of collagen I fibers within the Microatlas grids was quantified by means of Fourier components analysis.^37^ The Fourier angular spectrum was fit to a Gaussian whose full width at half maximum [FWHM, Fig. 5(j)] is taken as a measure of the collagen fibrils anisotropic distribution with respect to the edge of the Microatlas lattice. The FWHM of the orientation distribution in the Microatlas is 25°±1°[Fig. 5(j)], significantly lower than the values measured in control samples, 48°±2°, despite the fact that the collagen density in the implanted embryos was not significantly different than in the untreated embryos [Fig. 5(i)].

## III. DISCUSSION

In this work, we exploited a microgrid (Microatlas device) implanted in the chicken CAM and colonized by the cells and vessels of the host recipient, to quantify aspects of the FBR, by means of prolonged and repeated intravital microscopy observations. To do so, the microstructure must allow tissue and vessel reconstitution within it. However, our previous scientific efforts related to imaging the Microatlas device after its implantation in living chicken embryos,^23^ indicated that angiogenesis in the microgrid was hindered in microstructures with a cubic pore size of about $20\times 20\times 20\;\mu m^{3}$, even if host cell infiltration could occur physiologically. Here, by adopting a cubic pore size of $50\times 50\times 20\;\mu m^{3},$ we confirmed that the reduced neo-angiogenesis that we observed in previous work was due to the pore size. Indeed, the typical size of the microvessels in a chicken embryo lies in the range 40–100 μm.^28,38–40^ The translation of the technology presented here to higher level animals, such as rodents, should not be limited further by the microvessel size due to the low dependence ( $≅M^{\frac{1}{12}})$ of the vessel size on the animal mass M.^39^

By devising a variable geometry multi-spot fabrication setup, we could fabricate in a limited time ( ≅45 min) a complex implantable microstructured chip that had four scaffolds for the host organism tissue regeneration (Fig. 1). The structural stability of the Microatlas was sufficient to stand the mechanical stresses of the growing tissue during its implant in chicken embryos up to EID14 (i.e., 7 days after the implant). This result was achieved by inserting thicker elements in the structure, with a X-shaped cross section that reduced the level of autofluorescence to a level that did not interfere significantly with the autogenous signals from the tissue, allowing *ex vivo* and *in vivo* imaging (Fig. 2).

The highly porous lattice of the Microatlas scaffold clearly allowed the infiltration of cells (Fig. 2), vessels [Figs. 5(a) and 5(b)] and collagen fibers [Figs. 5(g) and 5(h)] in the structure, supporting the achievement of a mechanically guided FBR. By means of a direct and systematic comparison with histological images of H&E stained tissue sections, we showed that high resolution TPEF microscopy images allow a detailed analysis of the cell and nuclei conformation, both outside and inside the Microatlas. In this way, we developed a dataset of avian cells [Fig. 3(d)] that we used for segmentation on fluorescence and SHG *in vivo* of non-linear excitation images, providing us with high resolution cellular details. The conclusions that we can draw from this analysis are remarkably comparable to those obtained from the analysis of H&E-stained tissue sections, but without the necessity for time-consuming sample preparation (like tissue sectioning and H&E staining).

The dynamics of tissue regeneration is essential to characterize the effect of the implant. We characterized it in our living model exploiting *in vivo* fluorescence imaging. The physiological tissue density in the control regions was reached at EID10 [Fig. 2(e)], with non-significant variations up to EID14. In contrast, the infiltration in the Microatlas reached a tissue density comparable to the one of untreated embryos by EID11, increasing up to 1.8 times the density in the control regions at EID14, similarly to vessels that were denser in the Microatlas than in the control [Fig. 5(c)]. This result is in agreement with the histology and physiology of the host chicken embryo, whose angiogenesis is known to be very much responsive to foreign implant on the CAM.^41–43^ The slightly larger value of the cell density in the histological images compared to fluorescence images [Fig. 2(e1) compared to Figs. 2(e2) and 2(e3)], can be ascribed to the fact that in the first case we did not have access to the Microatlas and we were therefore probing the tissue in close vicinity to it. This effect is more evident for the granulocyte subpopulation [Fig. 4(i)] that was found within the Microatlas with ≃ 3.3 times lower density than outside. However, both histological and fluorescence images provided comparable values of the relative increase in the granulocytes in the samples implanted with Microatlas, with respect to the control samples [Fig. 2(e3)]. In the control samples, we confirmed on the histopathological images [Fig. 3(a) and Figs. SI3.1 and SI3.2] the presence of a wide population of cells, localized in the chorion layer and characterized by nuclei with shapes of varying eccentricity [Figs. 4(g) and 4(h)]. We found nucleated red blood cells in the vessels [Fig. 3(a3) and Figs. 3(c2) and 3(c4)], and other types of cells (i.e., fibroblasts) in the dense mesoderm, in which secretory vesicles were also visible (Fig. SI3.1), likely with the function of calcium provision to the embryo.

Regarding the reaction of the embryo to the implant of the Microatlas, it is known that in chicken embryos, as well as in other vertebrates, granulation tissue develops when the acute and chronic inflammatory responses are extinguished. On microscopy images we can identify granulocytes, fibroblasts, collagen and an increase in the neo-vascularization rate.^5^ Indeed, SHG imaging revealed the presence of fibrillar collagen I, which was an indication of an ongoing fibrous scarring process. Collagen I was found to be as dense in the Microatlas as in the control tissue, even though it appeared wrapped around in control tissue with a high angular dispersion of the fibers ≅ 50°, while it appeared more oriented, with an angular dispersion ≅ 25°, within the scaffold trusses [Fig. 5(j)]. As a matter of fact, extra-cellular matrix and collagen I production from fibroblasts invading a scaffold are known to depend both on the physical and chemical properties of the scaffold material^44–46^ and on the scaffold geometry and size^47–49^ (i.e., fiber diameter or thickness, as in our case). Apart from these findings, we had no evidence of a fibrotic capsule comprising thick, densely packed bundles of collagen fibers that may hinder the diffusion of analytes into the microstructure,^50^ possibly leading to chronic inflammation states and to infections.^5,46^ Instead, our observations point toward a wound healing process, with a temporal progression of inflammatory states.^51^ Moreover, the presence of micro vessels regenerated within the Microatlas scaffolds would likely ensure an appropriate access to the inner pores of the Microatlas to small molecules to which the vessels are permeable.

By comparing the microvascular network regenerated in the Microatlas with the ones reported in the literature for similar imaging windows or scaffolds,^9,17,23^ we can confirm that a porous structure with pore size of about 50 μm, as the one used here, allows angiogenesis [Figs. 3(c) and 3(d), Figs. 4(a) and 4(b), Fig. 5(a)]. Indeed, apart from the increase in the vessel size around the site of the implant [Figs. 2(f) and SI3.2] due to hyperemia, we could observe neovascularization inside the Microatlas occurring primarily through small capillaries [Figs. 5(e) and 5(f)]. The Microatlas scaffolds enhanced the neo angiogenesis *in situ*, a strategy that could allow the regeneration of a functional microvascular network in close contact to a biomaterial coupled to the Microatlas. This outcome would let analytes diffuse into the device, with a minor impact of the overall collagen capsule thickness, which is however limited around and within the Microatlas [Fig. 5(g)]. This result is particularly relevant since the vascularization of an implanted device is a preliminary condition to ensure a limited inflammatory reaction by the host.

As a result, we may presume that the formation of fibrotic tissue around the device is mild enough to allow trans-capsular moieties diffusion, as suggested by previous works on porous biomaterial implantation.^50–52^ To confirm this deduction, we will perform additional studies on the activity of the cells within the Microatlas. In fact, specific geometrical features in the substrates (such as channel width, pore size, etc.) could play important roles in the way fibroblasts orient and attach to the substrate,^5,53–56^ especially by controlling cell metabolic activity,^5,57^ and the interaction of the microstructure with other types of cells, like endothelial cells and macrophages.^53,58^ We also plan to translate these studies to other animal models with more developed and known immune systems. In the chick embryo, in fact, the possibility to differentiate among different types of immune cells is limited, to our knowledge, to the monoclonal antibody *KUL01* that label mononuclear phagocyte cells like granulocytes, monocytes or macrophages, differentiating them from T and B lymphocytes.^59^ We could, in this way, determine whether T and B cells are recruited at the implant at later stages (i.e., EID14) when granulocytes recruitment is already fading. However, macrophages of different phenotypes, at least in the two wide classes of pro-inflammatory or pro-healing cells, cannot be tracked due to the absence of specific antibodies, which are not yet available for this species.

The absence of the formation of a thick fibrotic capsule indicated a limited specific reaction due to the resin used for the fabrication of the microstructures. This could also be partially due to the fact that at the considered developing stages, chicken embryos immunocompetent system is not fully developed having rejection conditions not established yet.^60^ As a matter of fact, chicken embryos are protected by an effective immune system composed of both B and T cells, which control the antibody and cell-mediated immunity, respectively.^61,62^ However, until EID 10 the chicken embryo immune system cannot be considered completely developed: the presence of T cells can be first detected at EID 11, but B cells appear only at EID 12.^63^ After EID 15, B cell begins to diversify but chicken embryos do not become immunocompetent until EID 18.^61^

Even by basing on label-free images only, we can draw some relevant conclusions on immune cell recruitment. We observed that the density of granulocytes near to the Microatlas implanted in CAMs (i.e., within 200 μm from the Microatlas) was 6.3±1.5 times at EID14, compared to control embryos [Fig. 4(i)]. Also, within the Microatlas, the density of the granulocytes was significantly greater than in controls, reaching a value of 3.3±0.3 times the value found in control CAM samples. These findings are particularly relevant if one also considers the reduction due to the steric hindrance due to the excluded volume given by the pillars of the lattice and indicate that the Microatlas can be efficiently colonized by the host cells. Similarly, we found that the granulocyte concentration inside the vessels, being their primary route of recruitment, was 4.2±0.5 times greater in the Microatlas implants than in the CAM membranes at EID14 [Fig. 4(j)]. Altogether, the limited deposition of collagen around the Microatlas, the high concentration of microvessels within the Microatlas, and the level of concentration of granulocytes indicate that, although a reaction is present, no acute inflammation is activated until EID14 in the implanted embryos. This particularly relevant since the reactions of chicken embryo to foreign bodies are significantly faster, compared to what observed in adult rodents or humans. Fibrotic capsules have been reported to form after four days from implantation of several types of artificial materials, i.e., in an interval between EID 9 and EID13.^64^ Therefore, the microstructured device developed here can be successfully used as a reference frame for longitudinal observations in chicken embryos.

Most importantly, even without species-specific antibodies for immune cells, we were able to identify and segment individual granulocytes [Figs. 3(d) and 4(d)] in and around the Microatlas [Fig. 5(i)] and in vessels [Fig. 5(j)] based on the dataset of cells developed on the non-linear excitation and confocal fluorescence images [Fig. 3(d)] and systematically validated against the H&E histological analysis. This dataset could be used, once expanded to other types of immune cells in higher organisms and together with automatic feature recognition algorithms,^65^ to assess the amount of the different types of immune cells triggered by the reaction to an implant.

Regarding the possibility to carry out similar studies *in vivo* on a higher organisms, like rodents, in a minimal invasive way, one should resort to through-skin non-linear optical microscopy. In this case, tissue induced scattering and spherical aberrations would reduce the image signal/noise ratio. Alternative to non-linear excitation microscopy, one could exploit photoacoustic microscopy.^66–68^ This technique exploits 10 ns pulsed laser sources to penetrate in the tissue and recover the spatial information from ultrasounds generated by the thermo-acoustic shock wave in the tissue. It is however limited in spatial resolutions by the ultrasound frequency bandwidth,^69^ reaching typical values of about 20–100 μm, two orders of magnitude larger than the ones obtained with non-linear excitation microscopy.

High resolution ( ≃1 μm) optical microscopy in deep tissue can be obtained by physical corrections of the optical aberrations. Spherical aberrations could be corrected with conventional Zernike polynomials approaches.^70^ High order optical modes correction methods must be implemented to tackle with tissue induced scattering, as recently proposed by Papadopolous *et al.*^71^ and improved by May *et al.*^72^ However, this method of correction of the tissue scattering works on a very limited field of view, of the order of few Airy disks. Alternatively, one can resort to use the NIR-II optical window that spans the range of wavelengths 1000 nm ≤λ≤ 1700 nm,^73–75^ that features higher penetration depth and reduced tissue scattering, exploiting three-photon excitation fluorescence of red fluorescent proteins (dsRed and dTomato) and third harmonic generation (THG) scattering at 1700 nm.^74,76,77^ These or similar approaches could also be combined with the use of implantable micro-lenses coupled to the Microatlas as proposed recently.^78,79^ By taking advantage of these additional technological advances, one can envision to extend the potential application of an imaging window, like the Microatlas presented here, to perform a full characterization of the immune reaction to the implant *in vivo* also in rodents.

## IV. CONCLUSIONS

We developed and characterized a two-photon polymerized miniaturized implantable imaging window, the Microatlas, and tested the possibility to use it to recapitulate accurately the reaction of a living organism to the implant of an exogenous material. To this purpose, we implanted the Microatlas in a model of a living system, the chicken embryo, in which we could demonstrate its capability in stimulating cell infiltration and neovascularization with no massive deposition of collagen up to seven days of implantation, a duration of implant at which, in chicken embryos, foreign body reactions are already overcome the acute phase.^64^

Validation of biomaterials for clinical use stems from the application of the ISO 10993 norm, that requires a simple classification of the extent of the capillaries proliferation in three classes: minimal, broad and extended (Table E.II in Ref. 4). However, not much attention is given to the size, the shape (ramification) and the state of the endothelium of the vessels grown around or infiltrated within the biomaterial. Most immunological response mechanisms (such as inflammation, allograft and xenograft preservation/reperfusion and rejection) are reflected by primary manifestations at the level of the microcirculatory system. Moreover, the successful application of regenerated tissues or even homologous implants, critically relies on the capability to elicit an effective and functional vascularization when embedded into the surrounding host tissue.^80,81^

It is then particularly relevant that we could exploit non-linear excitation imaging (TPEF and SHG) to develop a high resolution cell atlas that allows to quantify *in vivo* and *ex vivo* the reactions occurring inside MicroAtlas grids at different time points with no need of specific markers (label-free). In this way we could single out granulocytes from the tissue, quantify them in the control samples, close to (within 200 μm) and in the Microatlas, as well as in the microvessels, and evaluate the density of collagen.

Our next steps will be to integrate different materials with the Microatlas and implant the combination of the two in animal models to observe the immune cell recruitment at the biomaterial surface. With the purpose to use this technology, i.e., digital pathology of the biomaterial immune reaction based on fluorescence microscopy, for biomaterial validation as per ISO10993-6 norms, we will elaborate protocols for the implant and observation in rodents.

Regarding the effective possibility to replace the standard histological analysis with non-linear excitation microscopy on microstructures guiding the reconstitution of tissue, one should consider that even if 2PP lithography is a costly and low throughput technology, the largest fraction of the cost of the tissue analysis with the non-linear excitation imaging on microstructures is given by the cost of the technician for the microscopy experiments. The cost of the microstructures for a massive production (about 180 000 pieces per year) is only 0.5 Euro per piece. The 3D optical sectioning by means of non-linear excitation microscopy allows to sample about 0.3 $mm^{3}$ of tissue per day. We then estimate a cost of 1100 €/ $mm^{3}$ for the non-linear microscopy analysis. The cost of conventional histopathology is again mostly determined by the cost of the technician. The volume of tissue sampled per day is limited by the number of stained tissue slices per day and by the thickness of the sections (typically μm). All together, we can estimate a cost of about 950 Euro/ $mm^{3}$ of sampled tissue. The two approaches are therefore comparable in terms of costs.

In conclusion, from the bio-engineering point of view, our results pave the way to a production of microfabricated structures for extended testing of implanted biomaterials. From the biological point of view, our results demonstrate the possibility to follow, in longitudinal studies, the immunological reaction to the implant based only on optical microscopy, opening the possibility of a direct monitoring of the reaction of the host animal to the implant *in vivo*. In addition, the potentiality of Microatlas proven here on chicken embryos, which is a simple animal model, its use in tissue imaging under high order nonlinear excitation (like three photons excitation and third harmonic generation), could allow to perform longitudinal studies of the foreign-body response also in higher animals, such as rodents.

## V. METHODS

### A. Samples preparation

#### 1. Sample preparation for two-photon laser polymerization

The photoresist employed for 2PP was the SZ2080,^24^ a negative organic–inorganic biocompatible resin, extensively validated for cell culture. It is made of two components: methacryloxypropil trimethoxysylane (MAPTMS, 97%, Sigma-Aldrich) and zirconium propoxide (ZPO, 70% in propanol, Sigma-Aldrich). ZPO enhances the material's mechanical stability. 1% wt. Irgacure 369 (IRG, Sigma-Aldrich) is added as a photo-initiator. The mass density as measured on a bulk polymerized specimen^82^ is 1200 kg/m^3^. The molar concentration of Irg369 is about 33 μM.

About 35 *μ*l of SZ2080 photoresist were deposited by drop casting on a 12 mm diameter circular glass coverslip (#1.5, Bio-Optica, Italy). The operation was always performed leaving a free external annulus on the glass substrate. This annulus has the role of assuring the correct holding inside the support. The solvent was removed through an evaporation phase (i.e., baking procedure) which occurred under chemical hood for at least 48 h at room temperature. Then, the resist reached a sol-gel state and allowed creating the initial chemical bonds between monomers and oligomers of the photoresist, preparing a starting substrate for the following laser-induced cross-linking.

#### 2. The chick-embryo implantation and preparation for microscopy

##### a. Implantation

Groups of 12–24 fertilized eggs were collected per week from a local farm (L'orto in casa ss., Correzzana, Italy) and stored in a dark cold room at the temperature of 10–15 °C. The eggs were dry washed with a brush and then stored in a clean egg-cup, blunt-end down. Fertilized chicken eggs can be stored at ∼13 °C up to 5 days before incubation without initiating development with a negligible degradation.^83^ Twelve eggs were incubated per experiment in a programmed egg incubator MG50 JR (FIEM, Italy), with serial automatic turning of eggs by an adjustable grid. The turning was activated from the beginning of the process with a constant rate and the temperature was maintained at 37.7 °C. A suitable air exchange was assured through proper ventilation holes. An Arduino-UNO based (Arduino, Italy) humidity system was connected to the incubator to maintain the humidity at 45% ± 5% during the *in ovo* cultivation. The *in ovo* incubation lasts 96 h. The Microatlas implantation *in ovo* occurred at the EID 7 *ex ovo* and was performed under a sterile cabinet. Control specimens (i.e., non-treated embryos) were implanted with a sterile circular glass coverslip, free from any microfabricated structures. For *ex vivo* measurements, at the end of the experiment (respectively, EID, 10–11–14) all the albumen content was removed and quickly substituted by formalin 4% (∼40 ml) assuring to completely cover the embryo. Then, the tissue portion was placed in a fridge at 4 °C to complete the fixing procedure, which lasted 72 h. Once formalin fixed, the embryo was washed three times in saline solution and stocked at 4 °C in Phosphate Buffered Saline (PBS). Both implanted and control regions were extracted after the washing procedure.

##### b. Ex vivo and in vivo imaging

*Ex vivo* samples analyzed by confocal fluorescence microscopy were processed to assure selective nuclei staining. Cell nuclei staining was performed following a previously defined protocol herein briefly summarized. The cellular membrane of dissected CAM portions was permeabilized in 0.25% v/v of nonionic surfactant Triton-X-100 (Sigma Aldrich, USA) for 15 min. Then, each sample was gently washed three times in PBS and stained with far-red nucleus fluorescent probe: DRAQ5 (AB1084104, Abcam, Italy) (having excitation peak of 647 nm and emission spectrum between 665 and 681 nm) at a concentration 0.2% v/v for 10 min. Finally, the sample was mounted with 30 *μ*l of the embedding solution Mowiol 4–88 on rectangular microscopy glass (30 × 22 mm^2^, 1#, ThermoFisher, USA). The samples were then stored for 24 h at room temperature or 72 h at 4 °C.

For histopathological analysis, the membrane CAM was fixed in 4% PFA, stained with Hematoxylin and Eosin, laid on a thin paper sheet and embedded in paraffin before being sliced along the cross section and along the plane of the membrane (4 μm section thickness).

The non-linear excitation microscopy experiments on chicken embryos *in vivo* were performed after the Microatlas implantation at the EID 7 on embryos *ex ovo*. A drop of PBS buffer was added on top of the embryo implant site to allow the image acquisition with a water immersion microscope objective. The embryo was kept in the thermostated box of the microscope that kept the sample stage at 37°C for the whole experiment.

### B. Optical setups

#### 1. Two-photon laser polymerization setup

2PP fabrication was performed by a laboratory-made femtosecond Ytterbium (Yb) - doped laser system, based on a cavity dumped mode-locked oscillator. The lasing wavelength was λ = 1042 nm, the pulse duration is ≅340 fs, the repetition rate 1 MHz, and the average maximum output power ≅8 W. The laser beam passed through a software controlled mechanical shutter (Uniblitz Electronics, LS Series, USA; maximum operating frequency ≅1 kHz) and was tightly focused by a plan-apochromat 100× oil immersion objective with numerical aperture (NA) 1.4 (Carl Zeiss, Germany) onto the photosensitive material, passing through the sample glass substrate.

A spatial light modulator (SLM) was introduced alongside the laser path and consisted of a driver unit with standard digital video interface (DVI or HDMI) and a phase only liquid crystal on silicon full HD micro-display. A Galilean beam expander, with a magnification of 5×, was used, before the SLM, to enlarge the beam (3.6 × 3.2 mm^2^) to completely illuminate all the surface of the SLM micro display (15.36 × 8.64 mm^2^). Finally, a Keplerian telescope, made as a series of two lenses (750 and 200 mm) (Thorlabs, USA), decreased the image size to fit the objective back projection.

In the Microatlas fabrication, only the first diffraction order was used while the others were blocked with an anodized aluminum slab.

SLM phase masks (400 × 400 pixels in size) were computed by the SLM Pattern Generator software, HOLOEYE. That software started from a binary image processed with the Gerchberg–Saxton iterative Fourier transform algorithm. By feeding computer generated holograms to the SLM display, we split the laser beam in multiple parallel ones with dynamically changeable positions and powers. The pixel pixel-micron conversion factor was experimentally determined along both X and Y directions observing an anisotropy due to the rectangular shape of the SLM screen,

$$\begin{array}{c} f_{x\;\text{axis}}=1.6\;\frac{\text{pixel}}{\mu m},\\ f_{y\;\text{axis}}=1.7\;\frac{\text{pixel}}{\mu m}. \end{array}$$ (1)

Therefore, aiming to a theoretical distance of 50 *μ*m, the corresponding pixel distance obtained was 31 pixels along the X axis and 29 pixels along the Y axis implying a mean line length along each direction approximately of 49.6 *μ*m.

The sample was mounted on an aluminum circular support connected to a gimbal mechanical system (Gimbal Mounts 100, Thorlabs, USA). The gimbal system presents an inner threaded hole, which allowed fabrication with various types of sample-holder, just fitting the cavity, giving the sample-holder a wide versatility. The sample holder-gimbal complex was mounted onto a planar (X, Y) brushless motion stage (ANT130XY Series, Aerotech, USA). The Z-direction, instead, was controlled by a motorized stage, balanced by two air compressed pneumatic pistons (ANT130LZS Series, Aerotech, USA), which counterbalance the gravity and avoid vibrations. These three stages were controlled via software (Automation 3200 CNC Operator Interface, Aerotech, USA) and equipped with a feedback position and velocity control system having a resolution on the order of nm. A red-light LED illumination was positioned under the sample-holder, in the central cavity of the gimbal, allowing the visualization during the writing process of the working area, as well as of the polymerized structures, through a CMOS camera (DCC1545M, Thorlabs, Germany). The three-axes stages and all the other set-up components were placed on a granite arch (ZALI, Precision granite technology, Italy), in turn placed above a pneumatic vibration isolator workbench (Newport, Stabilizer, High Performance Lamina Flow Isolator, I-2000 Series, USA).

After the laser fabrication process, the samples were developed to remove all the un-polymerized photoresist. Briefly, the sample glass surface was soaked for 25 min in a glass beaker filled with a 50% (v/v) 2-pentanone, 50% (v/v) isopropyl alcohol solution (Sigma-Aldrich, USA). Then, the samples were washed with abundant isopropyl alcohol and then gently dried by room temperature Nitrogen.

#### 2. Confocal microscopy

The Microatlas confocal fluorescence acquisitions were performed by a NIKON A1R or on a LEICA SP5 confocal microscope acquiring at 512 × 512 and 1024 × 1024 pixel^2^ resolution (spacing along the optical axis = 1 *μ*m per cell counting and 0.3 *μ*m per nuclei segmentation) using the 488 and 633 nm laser lines. On the Nikon microscope either a 20× dry NA = 0.8 or a 40× water immersion NA = 1.15 objective was used. On the Leica microscope, either a 20× dry NA = 0.5 or a 40× oil immersion NA = 1.3 were used. DRAQ5 was used for staining nuclei with an excitation wavelength of 633 nm and collecting light through an emission bandpass filter in the range 645–720 nm.

#### 3. Non-linear excitation microscopy

The Microatlas TPEF and SHG acquisitions were performed on custom setup based on a Ti:sapphire femtosecond laser (MaiTai Deepsee, Newport, USA) with pulse duration of ≃ 250 fs at the sample plane, having a tunable emission wavelength (690 nm < λ < 1020 nm). The beam passes through a commercial scan-head (FV300, Olympus, Japan) and reaches the BX51 Olympus upright optical microscope. The employed objective was a 25× water-matched (working distance 2 mm and NA 1.0, Olympus, Germany). Spectral separation of the emitted light was achieved by dichroic mirrors and bandpass filters in front of each photomultiplier tube (Hamamatsu H7422-40 for the 400/40 nm and 535/50 nm, 590/50 nm channels).^11^ The entire microscope was surrounded by a custom-made thermostatic cabinet in which the temperature was kept at 37 °C during *in vivo* inspections (Air thermostating by “The Cube,” Life Imaging Services, Basel, Switzerland). Multistacks were collected at 512 × 512 or 1024 × 1024 pixel resolution with an axial spacing along the optical axis of 1 *μ*m.

#### 4. Image processing and data analysis

##### a. Noise reduction on microscopy images

Confocal and TPEF images have been processed with Noise2Void (N2V), a recently published noise reduction deep learning method.^84^ This technique is able to mitigate all forms of non-structured noise, such as Gaussian and Poisson noise (signal fluctuations, shot noise, readout noise and quantization errors). The processing was performed on a desktop tower (Alienware Aurora R10, Dell, USA) equipped with a dedicated GPU (GeForce RTX 3090 & 24GB GDDR6X, NVIDIA, USA). All the acquired z-stacks were divided into groups characterized by the same imaging modality and experimental conditions (i.e., z-step and number of channels) in order to train each sub-set independently, while minimizing the number of required learning sessions. We employed a standard 3D-N2V configuration with U-Net depth = 2, kernel size = 3, and a mean square error (MSE) loss. The largest patch shape able to fit into memory was 64 × 64 × 8 × 1 pixels (XYZC). For each sub-set of images, a model was trained for 200 epochs (1–2 h) with batch normalization active. See also SI5, “Example of N2V Denoising on linear and non-linear images” for additional details.

##### b. Nuclei segmentation, cell density and anisotropy measurement

Cell nuclei visible in the processed fluorescence Z-stacks (as a positive signal on confocal images and as a negative signal on the non-linear excitation images) were 3D segmented by means of Imaris software (Oxford Instruments, UK) through the “magic wand” tool of the Surface View Mode. After segmentation of each nucleus, quantitative parameters were extracted such as surface area, volume, and ellipsoid axes. For the reported analysis, we mainly focused on the estimated ellipsoid axis since they provide a more unbiased representation when compared to volume or surface estimations, that are limited by the efficacy of the 3D reconstruction.

About 80 cells were manually segmented from fluorescence images taken on three implanted and three control areas. For cell density quantification, three different time points (day 3, day 4, and day 7 after implantation) were considered. The cell density of each sample (Microatlas and control) was calculated assuming a homogeneous distribution of the cells along the vertical coordinate as follows:

$$\mathrm{Cell}\;\text{density}=\frac{\mathrm{Cell}\;\text{number}}{\mathrm{Volume}}.$$ (2)

At least six random regions of interests (ROIs), 100 × 100 *μ*m^2^ in size were inspected per Microatlas and in the control samples. To assure the full 3D reconstruction of the cells in the volume, the height of the inspected volume varied in the range 20–45 *μ*m. The cells were quantified by using the ImageJ Multipoint tool, manually selecting each cell throughout the volume, and summing up the number of counts (cells) for each ROI.

##### c. Nuclei segmentation of cells in H&E histology images

The H&E-stained CAM sections were digitally scanned using a NanoZoomer-SQ (C13140) whole-slide scanner (Hamamatsu). The scanner captured images with a 40× dry objective (NA 0.75) and a spatial resolution of 220 nm/pixel (115454 dpi). Data were stored in NanoZoomer Digital Pathology Images (ndpi file format) using JPEG (Q = 80) compression scheme. Images have been manually annotated on QuPath.^85^ For the evaluation of the cell density on H&E images of the tissue sections, we ascribed to the tissue section the volume of the section (4 μm) plus the average cell size ( 5 μm).

##### d. Vessels' segmentation

The blood vessels were segmented and analyzed along the whole acquired thickness: 80–100 *μ*m per Microatlas, while the control samples were analyzed along the total CAM thickness (30–50 *μ*m). By using ImageJ ROI tool, vessels were manually segmented by following the capillary tube midline while computing the tube thickness (as the distance between the tube walls) every 100 *μ*m along the planar dimension and averaging all the data per vessel. Vessels whose length were lower than 100 *μ*m, comprised a thickness value obtained as the average of measures between the two extremities. Capillaries were inspected alongside different acquired stacks, assuring the uniqueness of each vessel between adjacent axial planes.

##### e. Second harmonic generation microscopy image analysis

*In vivo* non-linear excitation microscopy images were processed to quantify collagen I fibers directionality (by means of SHG) and blood vessels density (by means of autofluorescence signal). SHG multistacks were processed by the ImageJ Directionality plugin. Through this tool, the distribution of the orientation degree of collagen fibers present in the Region of interest was assessed per Microatlas pore. Signal from three sequential axial acquisitions were averaged and then the whole Microatlas height, ∼30% each of the total multistack, was arbitrary divided in three macro groups (LOW–MEDIUM–HIGH), ∼30 *μ*m each, starting from the glass interface, and the directionality averaged in those groups.

##### f. Collagen segmentation and quantification

TPEF and SHG acquisitions were performed on the same H&E-stained slides used for assessing the immunological response. Multistacks were collected using the non-linear excitation Olympus microscopy setup. The imaging was conducted at a resolution of 1024 × 1024 $\text{pixel}s^{2},$ with an axial spacing of 2 μm along the optical axis. Tile-scanning was employed to create a mosaic image of both EID14 and control samples.

The collected stacks were analyzed using ImageJ software. Within each tile, we identified the most in-focus z-slices and summed their signals. A median filter with a radius of 2 pixels was applied to enhance contrast and reduce noise. The two-photon primed fluorescence of the H&E stain was used to estimate the total tissue area in terms of pixel count. Simultaneously, the SHG signal was manually thresholded to highlight the collagen-covered areas.

To compare the EID14 implanted sample with the control, we calculated the fraction of collagen relative to the total tissue area. This involved computing the ratio between collagen-positive pixels and total tissue pixels across 25 different tiles for each CAM sample. After conducting the analysis, no significant differences (P > 0.05) were observed between the implanted and control samples based on the results of the two-tailed unpaired t-test.

##### g. Statistical analysis

In all the presented analyses, Kolmogorov test was used to inspect normality distribution inside each macro group, one-way ANOVA to test significant differences between experimental groups, and once assessed the homogenous distribution, Mann–Whitney test was used to compare implanted and untreated groups. All the collected data were fit in Origin (Originlab, USA).

## SUPPLEMENTARY MATERIAL

See the supplementary material for Microatlas design, on the two-photon laser polymerization setup and parallelization of the fabrication, on the histopathological analysis of CAM, on the analysis of the shape anisotropy of cell nuclei in CAM. Finally, details on the N2V denoising algorithm for linear and non-linear images.

## Data Availability

The data that support the findings of this study are available from the corresponding author upon reasonable request.
